# Modeling of Downlink Interference in Massive MIMO 5G Macro-Cell †

**DOI:** 10.3390/s21020597

**Published:** 2021-01-16

**Authors:** Kamil Bechta, Cezary Ziółkowski, Jan M. Kelner, Leszek Nowosielski

**Affiliations:** 1 Nokia Solutions and Networks, 54-130 Wrocław, Poland; kamil.bechta@nokia.com; 2 Institute of Communications Systems, Faculty of Electronics, Military University of Technology, 00-908 Warsaw, Poland; cezary.ziolkowski@wat.edu.pl (C.Z.); leszek.nowosielski@wat.edu.pl (L.N.)

**Keywords:** 5G, downlink, interference, signal-to-interference ratio (SIR), massive MIMO, multi-beam antenna system, multi-elliptical propagation model, 3GPP standard

## Abstract

Multi-beam antenna systems are the basic technology used in developing fifth-generation (5G) mobile communication systems. In practical implementations of 5G networks, different approaches are used to enable a massive multiple-input-multiple-output (mMIMO) technique, including a grid of beams, zero-forcing, or eigen-based beamforming. All of these methods aim to ensure sufficient angular separation between multiple beams that serve different users. Therefore, ensuring the accurate performance evaluation of a realistic 5G network is essential. It is particularly crucial from the perspective of mMIMO implementation feasibility in given radio channel conditions at the stage of network planning and optimization before commercial deployment begins. This paper presents a novel approach to assessing the impact of a multi-beam antenna system on an intra-cell interference level in a downlink, which is important for the accurate modeling and efficient usage of mMIMO in 5G cells. The presented analysis is based on geometric channel models that allow the trajectories of propagation paths to be mapped and, as a result, the angular power distribution of received signals. A multi-elliptical propagation model (MPM) is used and compared with simulation results obtained for a statistical channel model developed by the 3rd Generation Partnership Project (3GPP). Transmission characteristics of propagation environments such as power delay profile and antenna beam patterns define the geometric structure of the MPM. These characteristics were adopted based on the 3GPP standard. The obtained results show the possibility of using the presented novel MPM-based approach to model the required minimum separation angle between co-channel beams under line-of-sight (LOS) and non-LOS conditions, which allows mMIMO performance in 5G cells to be assessed. This statement is justified because for 80% of simulated samples of intra-cell signal-to-interference ratio (SIR), the difference between results obtained by the MPM and commonly used 3GPP channel model was within 2 dB or less for LOS conditions. Additionally, the MPM only needs a single instance of simulation, whereas the 3GPP channel model requires a time-consuming and computational power-consuming Monte Carlo simulation method. Simulation results of intra-cell SIR obtained this way by the MPM approach can be the basis for spectral efficiency maximization in mMIMO cells in 5G systems.

## 1. Introduction

Achieving greater transmission capacity for wireless links is the main goal of the currently developed fifth-generation (5G) mobile communication system [1,2]. The use of new spectral resources that cover frequency ranges exceeding 3 GHz provides an increase in the performance and capacity of next generation networks. However, propagation phenomena that occur in the centimeter-wave (cmWave) and millimeter-wave (mmWave) ranges cause numerous problems in the practical implementation of radio transmission equipment solutions [3,4]. The increase in propagation environment attenuation at higher frequencies makes it necessary to reduce the size of cells and sectors served by individual network base stations (BS). Hence, obtaining full coverage forces increased density of BSs in a given deployment area. The dominant amount of mobile users’ equipment (UEs) of wireless networks occurs in urban areas, where the phenomenon of multipath propagation significantly limits the transmission capabilities of radio links. In combination with the Doppler effect, resulting from user motions, this phenomenon leads to signal dispersion in time, frequency, and reception angle domains [5].

A multi-antenna system is one of the basic solutions used in the currently implemented 5G systems that minimize adverse propagation phenomena. A massive multiple-input-multiple-output (mMIMO) technique plays a special role [6,7]. It uses a beamforming technique [8,9], which allows for the possibility of practical implementation of spatial multiplexing for radio resources. This multiplexing improves spectral efficiency by using the same frequency sub-bands in angularly separated beams (spatially orthogonal beams). In urban areas, multipath propagation is the cause of the angular dispersion of the received signals [10]. It is the reason for receiving signal components from unwanted beams that significantly interfere with the signal from the serving (i.e., useful, reference) beam. A level of this interference is directly connected with spatial orthogonality between reference and interfering beams. If a signal-to-interference ratio (SIR), i.e., the ratio between received powers of reference and interfering signals, is higher, then the spatial orthogonality between these beams is better. Therefore, at the mMIMO 5G network planning and optimization stages, it is important to assess spatial orthogonality in realistic propagation conditions accurately. This allows achievable performance for a given deployment scenario to be estimated. One of the metrics that can help estimate mMIMO cell performance is the relation between the SIR and angular separation between the reference and interfering beams. Since UEs are distributed mostly on a horizontal plane, rather than a vertical one, in the typical cells of a mobile network, in the majority of cases is enough to consider angular separation only on a horizontal plane to make an accurate estimation of the SIR. In other words, accurate modeling of the relation between the angular separation of reference and interfering beams on a horizontal plane and the SIR helps to estimate many parameters of mMIMO cells. For example, we may determine a minimum distance between UEs that can be served by simultaneous mMIMO beams with an assumed interference level or maximum number of uniformly distributed UEs, which can be served by simultaneous mMIMO beams with the assumed level of the SIR.

In the literature, an interference subject concerns interfering signals in a wide-sense. The nature of their formation may be diverse. In most cases, when we talk about interferences, we mean so-called non-intentional interferences, i.e., those arising from the operation of radio, electronic, or mechatronic devices, networks, or whole systems during their work. The second group is the so-called intentional interference (i.e., jamming) mainly used in the military or security to disrupt enemy communication systems or counteract radio systems in a protected area (i.e., electromagnetic curtain [11]), e.g., in airports, buildings, and infrastructure of strategic importance. Examples of jamming 5G systems are presented in [12,13]. The remainder of the paper focuses on non-intentional interferences.

The interference subject in communication systems, in particular in 5G systems, is widely represented in the literature. Works in this area focus on the three following topics:

- interference cancelation, mitigation, awareness, and management methods,
- interference modeling and assessment methods,
- interference estimation and measurement methods.

Software-based algorithms and hardware solutions that are implemented in BS and UE belong to the first method group. Papers focusing on this topic present novel solutions, usually based on simulation analysis, e.g., [14,15,16,17,18,19]. The purpose of these methods is to increase the efficiency of devices and networks and make better use of radio resources.

Various modeling methods are used in interference evaluation. They are usually based on energetic assessment of the received signals. However, interference level analysis may take different aspects into account. In this case, the important aspects influencing the received signal form have a crucial value in the faithful reflection of the modeled issue in relation to the real situation. These aspects include, first of all, the channel model, as well as the parameters and characteristics of antenna systems. The possibility of considering environment nature and propagation conditions, as well as the appropriate reflection of angular dispersions affecting the received signal powers, should be taken into account when choosing a channel model. On the other hand, considering the parameters and patterns of antenna systems is of crucial importance, especially in the analysis of 5G spatially multiplexing systems, including those ensuring beamforming (e.g., with the mMIMO system).

Modeling methods are used to evaluate existing systems or new solutions (e.g., new mitigation algorithm) and, in particular, to evaluate inter- and intra-system electromagnetic compatibility, coexistence of 5G with other systems (e.g., fixed satellite services (FSS) [20,21], radars [22], long term evolution (LTE) [23], etc.) or to assess 5G network/system efficiency under occurring interference [24]. For 5G systems, intra-system interference (also called self-interference) analysis concerns, i.e., inter-cell [16,17,18,19] or inter-beam (or intra-cell) [17,24] interferences. In this case, we would like to note that most of the works available in the literature focus on inter-cell rather than inter-beam interference analysis. These methods are usually used in the network design and planning stages. This paper focuses on this group method for modeling and evaluating inter-beam interference in 5G massive-MIMO systems.

The last group of research and scientific works focuses on interference measurements in real environments for existing systems and networks, e.g., [23,25,26].

In this paper, we present a novel approach for assessing the interference level in a downlink (DL) that arises as a result of using a multi-beam antenna system in 5G BS (gNodeB), which is based on a multi-elliptical propagation model (MPM) [27]. Simulation results of the DL SIR obtained with the use of the MPM were compared with simulation results of the commonly used 3rd Generation Partnership Project (3GPP) channel model [28]. Simulations have been performed for realistic beam patterns of mMIMO antenna systems [29] and parameters of 5G networks determined by the 3GPP and International Telecommunication Union (ITU) [30]. These assumptions indicate the originality of the obtained results and the MPM approach for determining the interference level from undesirable beams, i.e., interfering beams of the antenna system.

Joint modeling of beamforming and angular spread is required to obtain an accurate estimation of realistic interference levels. Used spatial filtering of multipath components by the antenna pattern is sensitive to time-variant radio channel conditions. Such an approach to the modeling of 5G systems performance is gaining more attention. For example, in [31], the results of link budget calculations in the real propagation environment of the mmWave system can be found, whereas the corresponding impact on the efficiency of antenna array tapering is described in [32]. The study presented in this article follows the same modeling principles. Therefore, it can be considered as valuable input to the current state of the art.

The rest of the paper is organized as follows. Section 2 describes practical ways of using multi-beam antenna systems. Section 3 presents the basis for assessing the interference level in the DL based on the use of the MPM and 3GPP channel model. Assumptions, obtained results, and conclusions from the performed simulations are presented in Section 4 and Section 5, respectively.

## 2. Multi-Beam Antenna System—Practical Aspects

One of the key differentiators of 5G is the ability to utilize the benefits of the mMIMO technique, especially the simplification of multiple-user access [1,2]. Due to a large number of antenna elements connected to multiple transmission-reception radio chains, fast fading, as seen by the gNodeB, gradually disappears, and the radio channel becomes flat in the frequency domain. This effect, called channel hardening, causes that in orthogonal frequency division multiplexing (OFDM) access each subcarrier has a similar channel gain. Therefore, different UEs from the same cell can be allocated to the whole available frequency bandwidth [6,7].

On top of this, mMIMO allows cell capacity in reference to conventional MIMO to increase significantly. Due to the spatial multiplexing of available resources obtained through energy focusing using large antenna arrays, i.e., beamforming, mMIMO allows the same frequency bandwidth to be reused by multiple UEs at the same time. However, such a multi-user scenario is only possible in the case of favorable propagation conditions, i.e., when propagation channel responses from the gNodeB are sufficiently different to simultaneously serve UEs (UEs are considered to be spatially orthogonal). From this viewpoint, the number of available resources in the cell are multiplied by the number of UEs. In less favorable propagation conditions, i.e., when the spatial orthogonality between UEs is not sufficient, the available radio resources have to be appropriately distributed. Usually, if different UEs are served by other beams, they can be allocated with full available bandwidth in different time slots to avoid intra-cell interference. In cases where the same beam serves multiple UEs, the available bandwidth is split between these UEs accordingly. It may also be possible that only a single UE will be under the coverage of two neighboring beams. This would result in a doubling of the resources available from a single beam, i.e., UE can be served in two consecutive time slots.

Even though, due to beamforming, mMIMO significantly limits inter-cell interference in reference to legacy MIMO, the problem of unavoidable re-use of training sequences, i.e., pilot contamination, by UEs in different cells still exists, and the inter-cell interference grows along with the number of base stations in the network [19]. Therefore, it is crucial that inter-cell interference, on top of intra-cell interference, is accurately modeled in the network planning and optimization stages, as well as accurately estimated and limited during network operation through sufficient precoding.

## 3. Interference Evaluation in Downlink

### 3.1. Fundamentals of the Multi-Elliptical Propagation Model

Dispersion in the angular domain is characteristic of areas where multipath propagation occurs, e.g., urbanized areas with non-line-of-sight (NLOS) or even line-of-sight (LOS) conditions [10]. In such propagation environments, the basis for power assessment is a power angular spectrum (PAS), p(θ,ϕ,D), where θ and ϕ are the angles of arrival (AOA) in the elevation and azimuth planes, respectively, and D is the distance between a transmitter (Tx) and receiver (Rx). This function allows the received power $P_{R}\left(D\right)$ to be determined according to the relationship [33]

(1) $$P_{R}\left(D\right)=\iint_{\Omega }p\left(\theta ,\phi ,D\right)d\theta d\phi .$$

where Ω={(θ,ϕ):θ∈[0°,90°),ϕ∈[−180°,180°)}.

Thus, knowing the PAS for signals for useful (i.e., reference, serving) and unwanted (i.e., interfering) beams allows the energy relation between them to be assessed. In this paper, we analyze the transmission of signals in a frequency range from 3 to 4 GHz and with receiving point distances at 100, 200, and 500 m. For these conditions, we can assume that the dispersion phenomenon of the received power dominates in the azimuth plane. This fact is shown in [27,33]. In this case, the SIR between the useful signal strength $P_{R0}\left(D\right)$ and the power of the interfering signal $P_{RI}\left(D\right)$ that comes from the unwanted beam has the following form [5]:

(2) $$SIR\left(D\right)\left(\mathrm{dB}\right)=10\log_{10}\frac{P_{R0}\left(D\right)}{P_{RI}\left(D\right)}=10\log_{10}\frac{\int_{-180{}^{\circ}}^{180{}^{\circ}}p_{0}\left(\phi ,D\right)d\phi }{\int_{-180{}^{\circ}}^{180{}^{\circ}}p_{I}\left(\phi ,D\right)d\phi },$$

where $p_{0}\left(\phi ,D\right)=\int_{0}^{90{}^{\circ}}p_{0}\left(\theta ,\phi ,D\right)d\theta$ and $p_{I}\left(\phi ,D\right)=\int_{0}^{90{}^{\circ}}p_{I}\left(\theta ,\phi ,D\right)d\theta$ represent the PASs of the serving and interfering signals in the azimuth plane, respectively.

**Figure 1:**
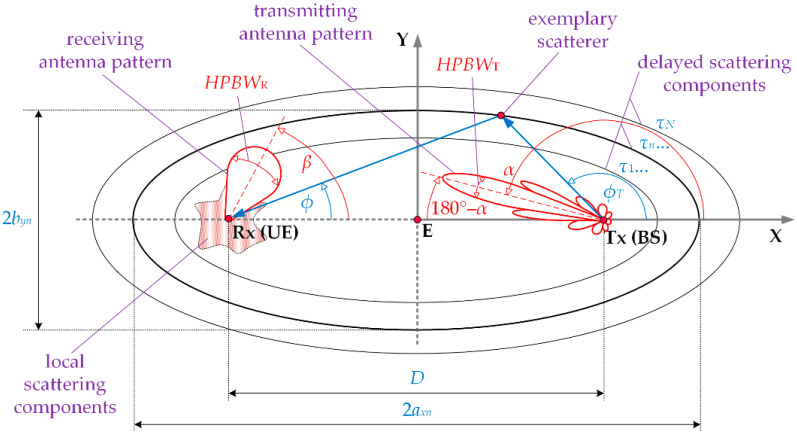
Scattering geometry of the multi-elliptical propagation model (MPM) in the azimuth plane.

**Figure 2:**
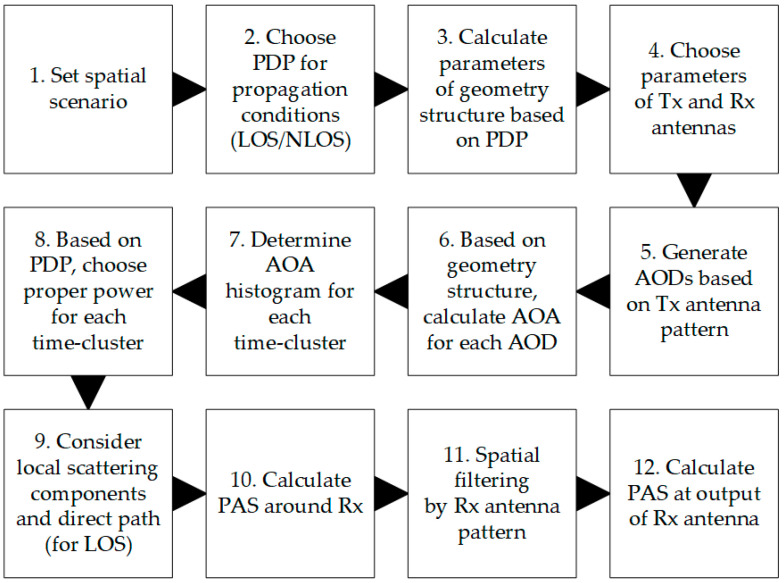
Simplified simulation procedure of the MPM.

Equation (2) reduces the SIR evaluation to determine $p_{0}\left(\phi ,D\right)$ and $p_{I}\left(\phi ,D\right)$ in the case when the MPM is used. The geometry of this model describes the most probable locations of scatterers. Its structure consists of a set of confocal ellipses whose foci determine the positions of the Tx and Rx, i.e., the gNodeB and UE for the DL scenario, respectively. The scattering geometry of the MPM in the azimuth plane is illustrated in Figure 1 [27], whereas Figure 2 depicts the simplified MPM simulation procedure.

Based on considered assumptions, i.e., the Tx-Rx distance—spatial scenario (step 1) and a chosen power delay profile (PDP) for LOS/NLOS conditions (step 2), in step 3, we calculate parameters of scattering geometry structure. For the *n*th ellipse (time-cluster), the major, $a_{xn},$ and minor, $b_{yn},$ axes are defined based on the PDP according to the following relationships [27,34]:

(3) $$a_{xn}=\frac{1}{2}\left(c\tau_{n}+D\right),$$

(4) $$b_{yn}=\frac{1}{2}\sqrt{c\tau_{n}\left(c\tau_{n}+2D\right)},$$

where c denotes the speed of light, $\tau_{n}$ is a delay for which the PDP takes the *n*th local extreme, n=1,2,…,N, and N is the number of time-clusters (i.e., the local extremes) in the PDP.

The adopted way of creating the MPM geometric structure enables mapping of the transmission properties of propagation environments. Detailed descriptions of this structure are provided in [27,33,34,35].

In step 4, we choose the Tx and Rx antenna parameters, i.e., their pattern shapes, gains, directions of maximum radiation/reception, and half-power-beamwidths (HPBWs). In the simulation testing procedure, the mapping of directional antennas is obtained using their normalized radiation pattern [35], which is realized in step 5. Since these characteristics meet the definition properties of probability density [36], in the simulation procedure, the directions of departure of propagation paths are generated on their basis. A detailed description of determining the radiation angle distribution is given in [35]. In step 6, based on the MPM geometry structure, AOAs are calculated for each angle of departure (AOD). The sets of the obtained AOAs for each time-cluster are the basis for determining the histograms in step 7. For each time-cluster, we choose appropriate powers defined in the analyzed PDP (step 8). Next, in step 10, we multiply the AOA histograms with the proper powers to obtain the PAS seen around the Rx [33,35]. At this stage, the local scattering components and direct path for LOS conditions are also considered (step 9). Using spatial filtering by the Rx antenna pattern, we calculate the PAS seen on this antenna output (steps 11 and 12) [33]. During interference analysis, we launch the MPM simulation procedure twice for the serving and interference Tx beams, respectively.

### 3.2. Fundamentals of 3GPP Channel Model

**Figure 3:**
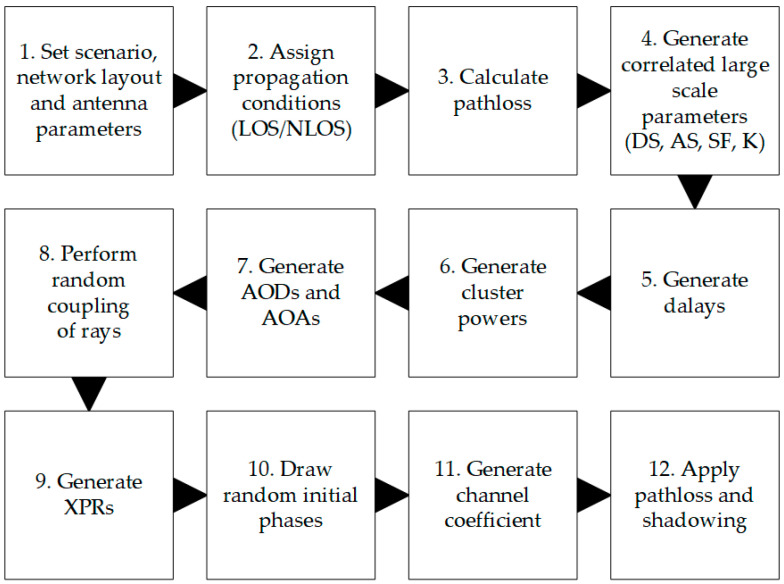
Block diagram of statistical channel model reconstruction according to 3GPP.

For link-level and detailed system-level simulations, the 3GPP has provided instructions on how to generate statistical three-dimensional (3D) channel models, as shown in Figure 3 [28]. It includes all the necessary radio propagation phenomena that must be considered during a comprehensive simulation to provide an estimation of the radio link budget (including interference) and performance.

It should be noted that according to [28], dispersion in the angular domain is modeled in steps 4, 6, 7, and 8 of Figure 3. In step 4, when the angular spread (AS) for a given scenario and network layout is generated, i.e., based on the assumed statistical model, the following parameters are generated:

- azimuth spread of departure (ASD),
- zenith (i.e., elevation) spread of departure (ZSD),
- azimuth spread of arrival (ASA),
- zenith spread of arrival (ZSA).

In step 6, the power for all rays of all clusters (which arise due to multipath propagation) is generated, whereas in step 7, the angles of departure and arrival are determined for all these rays. Finally, in step 8, random coupling is performed between departure and arrival angles for rays inside a given cluster, in both azimuth and elevation. As can be noticed, according to the 3GPP channel model [28], the PAS, p(θ,ϕ,D), obtained at the end of step 8 does not depend on the assumed antenna pattern. It is considered only in step 11, where channel coefficients for each cluster and each Tx and Rx element of antenna arrays are generated. Only at the end of step 11 are results of the spatial filtering of multipath components (clusters and rays) by the Tx and Rx nominal antenna patterns known. Therefore, to correctly calculate the received power of either reference or interfering signal, it is required to determine the effective antenna gains for the Tx and Rx. These effective antenna gains are defined as an integral part of the multiplied nominal antenna pattern (for the Tx or Rx) and PAS, which is equivalent to spatial filtering, as shown below [37]:

(5) $$G^{Eff}\left(D\right)=\iint_{\Omega }g^{Nom}\left(\theta ,\phi \right)p\left(\theta ,\phi ,D\right)d\theta d\phi ,$$

where $g^{Nom}\left(\theta ,\phi \right)$ indicates nominal 3D antenna pattern, either for the Tx or Rx, in either the reference or interfering link. Similarly, $G^{Eff}\left(D\right)$ indicates the effective gain of the Tx or Rx in either the reference or interfering link. Following the notation of Equation (2), the SIR calculated according to the 3GPP channel model [28] may be presented as follows:

(6) $$SIR\left(D\right)\left(\mathrm{dB}\right)=10\log_{10}\frac{P_{R0}\left(D\right)}{P_{RI}\left(D\right)}=10\log_{10}\frac{G_{T0}^{Eff}\left(D\right)\cdot G_{R0}^{Eff}\left(D\right)}{G_{TI}^{Eff}\left(D\right)\cdot G_{RI}^{Eff}\left(D\right)},$$

where $G_{T0}^{Eff}\left(D\right),$ $G_{R0}^{Eff}\left(D\right),$ $G_{TI}^{Eff}\left(D\right),$ and $G_{RI}^{Eff}\left(D\right)$ indicate the effective gains of the Tx and Rx in reference and interfering links, respectively.

## 4. Simulation Studies

### 4.1. Assumptions

**Figure 4:**
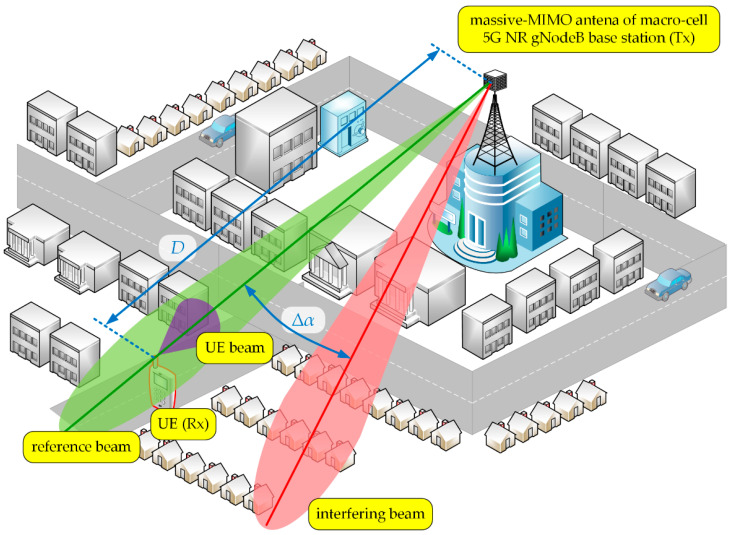
Spatial scenario of simulation studies [38].

In the simulation studies, we considered a scenario illustrated in Figure 4 [38]. In this case, the macro-cell gNodeB (Tx) with the mMIMO antenna array generates two beams in the selected sector, i.e., reference and interfering beams marked in green and red colors, respectively. Their directions determined the angle of beam separation, Δα. The UE (Rx) is in an area of the reference beam at distance D. Directions of the UE (purple color) and reference gNodeB beams provide their alignment. We assessed the DL SIR versus Δα between the serving and unwanted beams for various distances D in an urban macro (UMa) deployment scenario.

**Figure 5:**
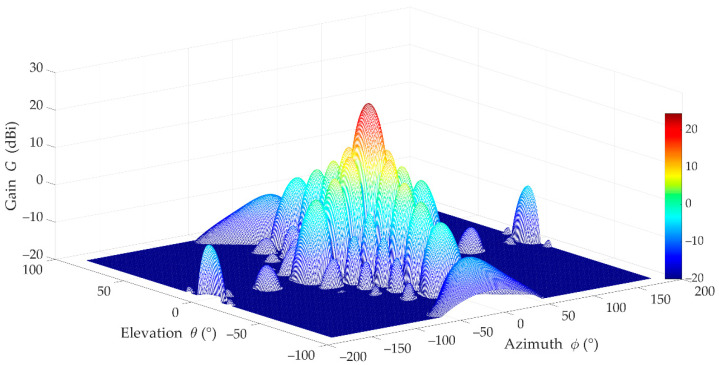
3D pattern of reference beam [38].

**Figure 6:**
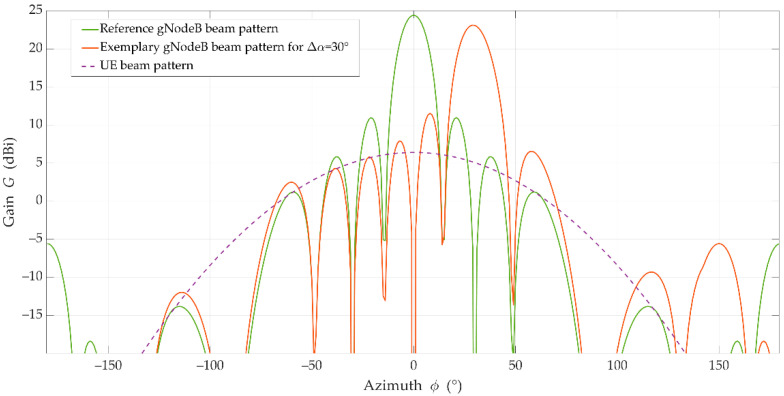
Patterns of users’ equipment (UE), reference, and exemplary interfering beams in the azimuth plane [38].

For more realistic results, we used practical patterns for the UE and gNodeB beams, and simulation assumptions developed by the 3GPP and ITU in [29,30]. The gNodeB was equipped with an antenna array of 8 × 8 elements that generate two analyzed beams in the selected sector. The UE beam with HPBWs equal to 90° and 65° on the horizontal and vertical planes, respectively, is generated by a single antenna element. Figure 5 depicts the 3D pattern of the reference beam [38]. The patterns of the UE (purple dashed line), reference (green line), and interference (red dotted line) beams in the azimuth plane are shown in Figure 6 [38]. In this case, the exemplary interfering beam is presented for Δα=30°.

The main simulation parameters are summarized in Table 1, whereas details of assumed channel models are as follows:

- in case of the MPM:
  o PDPs are based on tapped-delay line (TDL) models from the 3GPP standard [28] (pp. 77–78, Tables 7.7.2-2, 7.7.2-4), i.e., TDL-D and TDL-B for LOS and NLOS conditions, respectively;
  o these TDLs correspond an UMa scenario and normal-delay profile, i.e., rms delay spread (DS) is equal to $\sigma_{\tau }=363\;\mathrm{ns}$ [28] (pp. 80, Table 7.7.3-2);
  o in the TDL-D for LOS conditions, the Rician factor is defined as κ=13.3 dB [28] (pp. 78, Table 7.7.2-4);
  o local scattering described by the von Mises distribution [39] with an intensity coefficient equal to γ=60;
- in case of the 3GPP model:
  o New Radio (NR) UMa LOS and NLOS statistical channel models with parameters from [28] (Section 7.5);
  o Monte Carlo simulation methodology with 1000 repetitions of statistical channel model realizations.

The angular spread characteristics for the UMa channel are determined by the 3GPP [28] using the inverse Gaussian and Laplacian functions for the azimuth and zenith spreads, respectively. The mean and standard deviation values for these distributions are given in [28] (pp. 42–44, Table 7.5-6 Part-1). Therefore, the Monte Carlo simulation methodology is required to obtain a statistically representative set of results.

Two separate simulation tools have been used to obtain results for the MPM and 3GPP model. In the MPM’s case, we use our own simulator developed in a MATLAB environment. It is based on analysis of propagation paths between the Tx and Rx, scattered on the multi-elliptical geometry, according to the block diagram depicted in Figure 2. Since 2015, the MPM and its simulator are developing [27,34]. In the first version, isotropic/omnidirectional pattern antennas were considered [34]. Next, we introduced the Gaussian pattern for the transmitting [35] and then for the receiving antennas [33]. In this case, the transmitting pattern is used to determine the path direction probability. In contrast, the receiving pattern is using for spatial filtering of the paths reaching to the Rx, similarly to the 3GPP model. In the last version of the simulator used in this research, we replaced the Gaussian patterns with realistic patterns based on3GPP recommendation [30]. In this case, we use the same approach as in the second simulator for the 3GPP model. The MPM simulator was validated at every stage of its evolution. In many of our papers, we showed its verification based on measurement data and comparison with other propagation models, e.g., [34,35,40]. Generating a huge number of propagation paths, we obtained an average result for the MPM based on only one simulation run. In this case, analysis of the confidence intervals of the obtained results is not possible. To achieve this aim, we have to follow an approach similar to that used in the 3GPP simulator, i.e., we must run multiple simulations for a small number of random propagation paths in accordance with the Monte Carlo process. We want to highlight that the calculation time for the Monte Carlo approach is definitely shorter for the MPM than the 3GPP simulator.

Simulation results for the 3GPP channel model have been obtained by a proprietary system-level simulator, also implemented in a MATLAB environment according to the block diagram depicted in Figure 3. A crucial part of this simulator is the implementation of the full 3D statistical channel model, as defined by the 3GPP in [28]. From the perspective of the results presented in this paper, the essential parts of the simulator used are the antenna model and fast fading models, based on Sections 7.3 and 7.5 of [28], respectively. As a UMa scenario has been assumed in this study, the most important statistical parameters of angular spread can be found in Table 7.5-6 Part-1 of [28]. This simulator is maintained and used to provide system-level simulation results as contributions to current 3GPP standardization works, as well as research studies, e.g., [37].

### 4.2. Results for LOS Conditions

**Figure 7:**
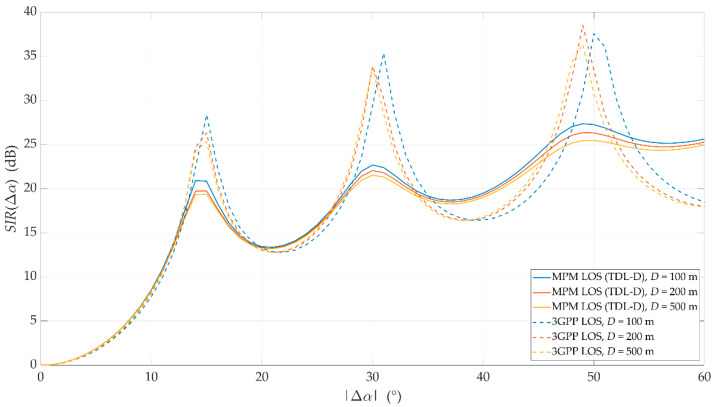
Signal-to-interference ratios (SIRs) versus angle of beam separation for line-of-sight (LOS) conditions and different UE–gNodeB distances obtained for the multi-elliptical propagation model (MPM) and 3GPP model.

**Figure 8:**
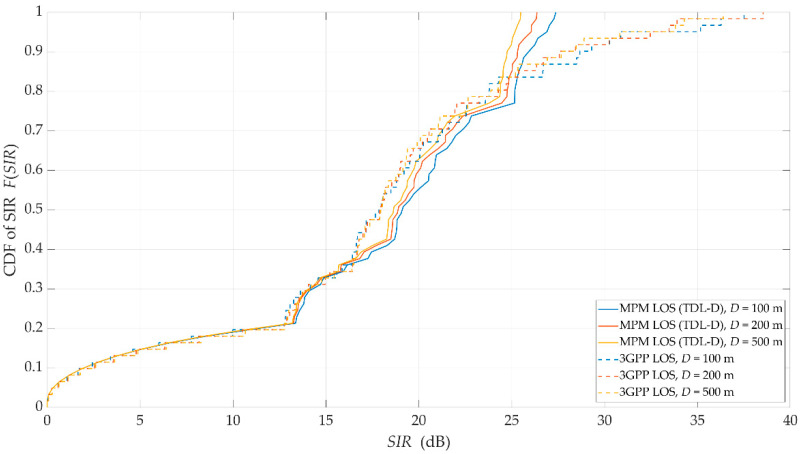
Cumulative distribution functions (CDFs) of SIR for LOS conditions and selected distances obtained for the MPM and 3GPP model.

For the above assumptions, we carried out simulation studies. Results obtained for LOS conditions are depicted in Figure 7 and Figure 8. Graphs in Figure 7 present the SIRs versus the angle of beam separation for the various distances between the UE and gNodeB, obtained for the MPM and 3GPP model. Figure 8 shows the corresponding cumulative distribution functions (CDFs) of SIR.

As can be expected, an increase in Δα reduces the DL interference between the reference beam (providing services to the UE) and the interference beam. However, the nature of the SIR graphs is not uniform. For Δα≅ 15°, 30°, and 48°, there are local maxima. They are visible for both assumed channel models and result from considering side lobes in the realistic patterns of gNodeB beams. For the 3GPP channel model, the magnitudes of local maxima are noticeably higher than in the MPM. High maxima in the 3GPP model are caused by the significant difference between powers of direct and reflected multipath components, which is typical for the 3GPP LOS channel models. However, for the remaining ranges of angular separation Δα, where local maxima are not present, the results obtained for the MPM and 3GPP model are comparable. The CDFs of SIR (see Figure 8) illustrate that for 80% of the analyzed range of angular separation, the results obtained by both models are within 2 dB, with half of these results within 1 dB of difference. This comparison of the LOS scenario clearly indicates that estimation of intra-cell interference and SIR with the use of the MPM demonstrates accuracy comparable with the 3GPP channel model.

To estimate intra-cell interference and SIR, the MPM only needs a single simulation instance. In contrast, the 3GPP statistical channel model requires computational power- and time-consuming Monte Carlo simulations. Taking the above facts into account, the MPM seems to be a reasonable alternative to the commonly used channel model of the 3GPP, especially if obtained simulation results are comparable.

### 4.3. Results for NLOS Conditions

Due to modeling of the transmission properties of the propagation environment, the 3GPP simulation test procedure is strictly statistical. On the other hand, the procedure used in the MPM is based on the PDP, which provides the creation of a geometric structure for the determined analysis of propagation paths. The DS ($\sigma_{\tau }=363\;\mathrm{ns}$) is the only joint parameter that describes the transmission properties of the environment. Under LOS conditions, the signal arriving at the Rx via the direct path is the dominant component of the received signal. Hence, according to 3GPP and MPM procedures, simulation tests give us results with a similar set of values and the nature of changes. Under NLOS conditions, the 3GPP approach gives a statistical estimate of the SIR as a function of the beam separation angle and the distance from the gNodeB. In contrast, the MPM procedure is associated with specific transmission properties of the propagation environment, which define the PDP and constitute one random set of parameters in the 3GPP procedure.

**Figure 9:**
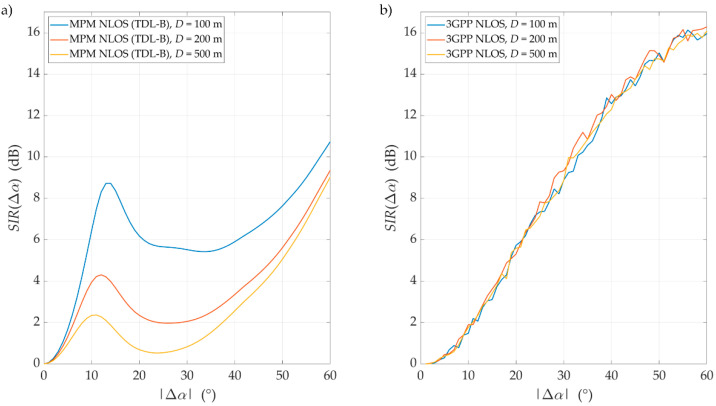
SIRs versus angle of beam separation for non-line-of-sight (NLOS) conditions and different distances between UE and gNodeB obtained for the (**a**) MPM and (**b**) 3GPP model.

**Figure 10:**
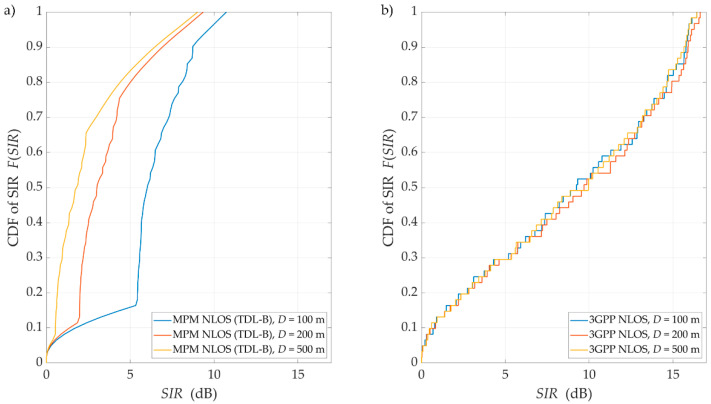
CDFs of SIR for NLOS conditions and selected distances obtained for (**a**) the MPM and (**b**) the 3GPP model.

For NLOS conditions, we carried out the simulation tests based on the assumptions described in Section 4.1. The obtained results are shown in Figure 9 and Figure 10. Charts in Figure 9 depict the SIRs versus Δα for various D obtained for (a) the MPM and (b) the 3GPP model, respectively. The corresponding CDFs of SIR are illustrated in Figure 10.

As can be expected, an increase in Δα reduces the DL interference between the reference beam (providing services to the UE) and the interference beam. However, the spreading effect of the propagation environment causes significant angular dispersion in the power of the received signals. This is the reason for an increase in the level of co-channel inter-beam interference, i.e., a decrease in the SIR by about 15 dB in reference to corresponding LOS results. The results obtained in the 3GPP simulation test process show little differentiation in relation to the EU position. This effect is due to the multiple uses of averaging in the 3GPP procedure. It consists of a random selection of the transmission parameters of the propagation environment in the next simulation step. The selection randomness of these parameters is limited only by the condition of ensuring a constant the DS value.

On the other hand, use of the MPM makes it possible to assess the impact of the UE position on SIR changes. In this case, the PDP unambiguously defines the transmission environment parameters that are the basis for determining the MPM spatial structure. Thanks to this, it is possible to map the Rx position in relation to scattering element locations, which determines the individual propagation path trajectories. The simulation test results clearly indicate that estimation of the intra-cell interference and SIR with MPM use demonstrates greater detail in comparison to the 3GPP channel model [27]. Therefore, the MPM seems to be a reasonable alternative to the commonly used 3GPP channel model, especially if we want to obtain an assessment for strictly determined PDP.

It should be highlighted that the 3GPP model is currently considered a standard in the analysis of 5G systems and beyond. However, this does not mean that the results obtained by this model always faithfully reflect the simulated scenario. This may be due to differences between the modeling approaches, i.e., statistical, stochastic, geometric, or deterministic. Many models in the literature also show divergence from the 3GPP model, e.g., [41,42,43,44]. The authors of [41,42] propose models based on empirical measurements carried out in Vienna and New York, respectively, whereas ray-tracing approaches are described in [43,44]. In the last, differences in the CDFs of the received interference power are shown.

### 4.4. Analysis of SIR Confidence Intervals

**Figure 11:**
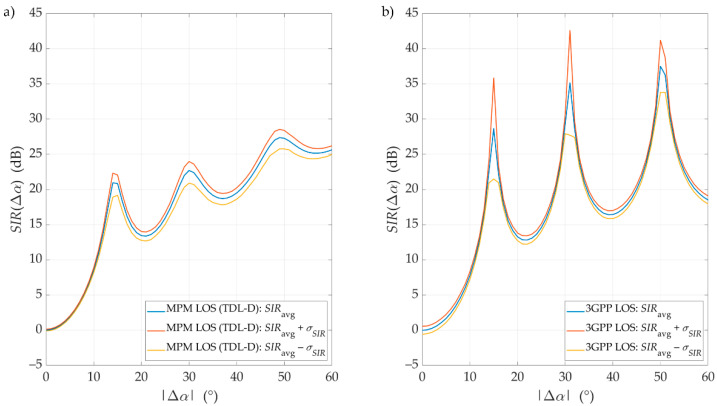
Exemplary SIRs with confidence intervals versus angle of beam separation for LOS conditions, *D* = 100 m, obtained for (**a**) the MPM and (**b**) the 3GPP model.

**Figure 12:**
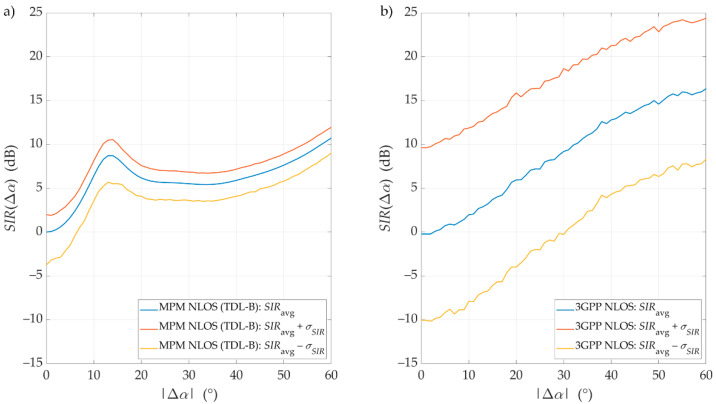
Exemplary SIRs with confidence intervals versus angle of beam separation for NLOS conditions, *D* = 100 m, obtained for (**a**) the MPM and (**b**) the 3GPP model.

To analyze simulation result variability, we determined the confidence intervals against the average SIRs, $SIR_{\mathrm{avg}},$ presented in Section 4.2 and Section 4.3. The obtained confidence intervals are depicted in Figure 11 and Figure 12 for LOS and NLOS conditions, respectively. In this case, considering the similarity of the results for various distances, we show the results only for D=100 m.

The confidence intervals of the SIR graphs were illustrated as $SIR_{\mathrm{avg}}\left(\Delta \alpha \right)\pm \sigma_{SIR}\left(\Delta \alpha \right),$ where $\sigma_{SIR}\left(\Delta \alpha \right)$ is a standard deviation for the selected separation angle, calculated based on 1000 values of the SIR set obtained during each Monte Carlo run. Using the Monte Carlo approach in the 3GPP simulator allows these confidential intervals to be obtained simultaneously. Average results for the MPM simulator, illustrated in previous sections, were obtained based on the so-called single-simulation mode. In this case, we used a huge number of propagation paths. To analyze the confidence intervals for these average results, we also had to run the MPM simulator in Monte Carlo mode with a small number of random propagation paths.

Generally, we may see that the MPM results fall within the ambit of the 3GPP confidence intervals. Under LOS conditions, both models’ results are very convergent, as described in detail in Section 4.2. The obtained results of the confidence intervals further increase their similarity. In this case, a characteristic feature is a slight increase in the deviation with the increase in Δα. Under NLOS conditions, the SIR results for the MPM fall wholly within the ambit of the confidence intervals obtained for the 3GPP model. For these propagation conditions, it is characteristic to keep the constant $\sigma_{SIR}$ regardless of the separation angle.

To compare error distribution of the simulation results, we calculated the mean standard deviation, $\bar{\sigma },$ for the MPM: ${\bar{\sigma }}_{LOS}^{MPM}=0.84\;\mathrm{dB}$ and ${\bar{\sigma }}_{NLOS}^{MPM}=1.83\;\mathrm{dB},$ and 3GPP: ${\bar{\sigma }}_{LOS}^{3GPP}=0.94\;\mathrm{dB}$ and ${\bar{\sigma }}_{NLOS}^{3GPP}=8.91\;\mathrm{dB},$ under LOS and NLOS conditions, respectively. In this case, SIR variability may be modeled as a Gaussian random variable with a standard deviation determined by the appropriate $\bar{\sigma }.$ Conversely, the average value of this random variable should be modeled using the averaged SIR described in Section 4.2 or Section 4.3. On the other hand, we can see the similarity of both simulators in their $\bar{\sigma }$ values for LOS conditions. Under NLOS conditions, the values of $\bar{\sigma }$ are definitely different. This may result from the fact that in the MPM, the scatterer locations are limited to the defined multi-elliptical structure related to the PDP. However, in the statistical 3GPP channel model, the potential positions of the scatterers are characterized by more spatial variation.

## 5. Conclusions

This paper focused on modeling the interference of radio DLs arising in multi-beam antenna systems, which helps to assess the performance of the mMIMO in 5G cells at network planning and optimization stages. Furthermore, we presented the comparison of two modeling methodologies that allow the DL intra-cell interference and SIR to be estimated. The presented methods of SIR evaluation were based on simulation studies. In this case, the MPM and 3GPP channel model, combined with realistic beam patterns and simulation parameters of the 3GPP/ITU, were used. The obtained results shown the effectiveness of the novel approach using the MPM in determining the minimum angular separation in multi-beam antenna arrays that provided an acceptable interference level compared with the simulation results obtained by the 3GPP channel model. Unlike the methods of inter-beam interference assessment used so far, the MPM solution proposed in this paper considers the phenomenon of the angular dispersion of received power. The ability to adapt the MPM geometric structure to actual transmission conditions minimizes SIR evaluation errors. In this, the presented novel MPM approach’s utilization for assessing the interference level at the receiving point could be considered as an efficient method for the determination of the required minimum angular separation between co-channel beams of mMIMO cells. Therefore, the MPM approach might help maximize spectral efficiency in 5G networks under deployment. This statement is justified as for 80% of simulated samples of the intra-cell SIR the difference between results obtained by the MPM and commonly used 3GPP model was within 2 dB or less for LOS conditions of the UMa network operating in a 3.5 GHz band. In the case of NLOS, the difference between both channel models is more visible. This may result from the fact that in the MPM, the scatterer locations are limited to the defined multi-elliptical structure related to the PDP. Conversely, in the statistical 3GPP channel model, the potential positions of the scatterers are characterized by more spatial variation. Further studies are being conducted in which the MPM effectiveness is assessed in mmWave simulation scenarios with both DL and uplink.

## Figures and Tables

**Table 1:** Main simulation parameters.

Parameter	Value
carrier frequency	3.5 GHz
distance *D* between gNodeB (Tx) and UE (Rx)	{100, 200, 500} m
height of gNodeB (Tx) antenna	25 m
height of UE (Rx) antenna	1.5 m
gain of single antenna element	6.4 dBi
HPBW of single antenna element	90° for H, 65° for V
spacing between antenna elements	0.5 of wavelength for H, 0.7 of wavelength for V
front to back ratio	30 dB
antenna array of gNodeB (Tx)	8 × 8
antenna array for UE (Rx)	1 × 1
range of angular separation Δ*α* in horizontal plane between reference and interfering beams	from 0° to 60°, with step of 1°

## Data Availability

The data presented in this study are available on request from the corresponding author. The data are not publicly available due to project restrictions.
